# Beyond Prediction: Integrating Biomarkers with Preventive Strategies to Shape the Future of Cardiac Surgery-Associated Acute Kidney Injury Management

**DOI:** 10.21470/1678-9741-2025-0375

**Published:** 2026-09-10

**Authors:** Khaled Alebrahim

**Affiliations:** 1 King Abdulaziz University, Alnaseem, Jeddah, Saudi Arabia. E-mail: dr.k.ebrahim@gmail.com

To the Editor,

We read with great interest the article by Zhang et al.^[^1^]^ in the Brazilian Journal of Cardiovascular Surgery entitled “Predictive Biomarker for Cardiac Surgery-Associated Acute Kidney Injury: A Retrospective Analysis.”. The authors demonstrated that tumor necrosis factor-α, interleukin-2, interleukin-6, and neutrophil gelatinase-associated lipocalin are significantly elevated in patients developing cardiac surgery-associated acute kidney injury (CSA-AKI), and that a composite biomarker panel achieved excellent predictive performance. This study highlights the promise of biomarker-based models in the early detection of CSA-AKI. We would like to share the findings in our earlier publication “Cardiac Surgery-Associated Acute Kidney Injury in Adults and Pediatrics; Prevention is the Optimal Management”^[^2^,^3^]^ which was overlooked. Our cohort of 941 patients, both adults and pediatrics, confirmed CSA-AKI incidences of 28.7% and 20.1%, respectively. Most importantly, our conclusion emphasized that prevention remains the cornerstone of CSA-AKI management. In that article, we provided a practical framework of preventive strategies, which included preoperative measures such as optimization of renal function, rehydration, and avoidance of nephrotoxic drugs; intraoperative approaches including minimizing cardiopulmonary bypass and aortic cross-clamping times, maintaining sufficient perfusion pressure, and balanced fluid therapy; and postoperative measures, mainly, tight glycemic control, careful hemodynamic monitoring, and early initiation of renal replacement therapy when indicated.

While biomarkers provide early risk identification, they do not in themselves prevent CSA-AKI. True clinical benefit arises when predictive signals are coupled with actionable preventive measures. Recent evidence reinforces this integration. The Kidney Disease: Improving Global Outcomes (or KDIGO) focuses on hemodynamic optimization, avoidance of nephrotoxins, glycemic control, and timely renal support, which has been shown to significantly reduce the incidence of CSA-AKI in biomarker-identified high-risk patients^[^4^]^. In addition, novel biomarkers such as TIMP-2*IGFBP7 and cystatin C, along with machine learning models integrating clinical and biomarker data, represent promising advances. Yet, the impact of these innovations depends on their implementation within structured preventive protocols. The integration of biomarker-guided identification with comprehensive preventive strategies offers the most effective pathway to reduce the burden of CSA-AKI and improve patient outcomes.

## Artificial Intelligence Usage

The author declares use of ChatGPT for grammar verification. The content produced by the artificial intelligence tool was revised and edited by the author as necessary, and the author takes full responsibility for the content to be published.
